# Editorial: Engineered extracellular vesicles for tissue repairing

**DOI:** 10.3389/fbioe.2023.1257165

**Published:** 2023-07-25

**Authors:** Shiyi Yu, Guoyong Chen, Siim Pauklin, Yunjiao Zhang, Yuliang Feng, Hao Chen

**Affiliations:** ^1^ Department of Gastroenterology, Guangdong Provincial People’s Hospital, Guangdong Academy of Medical Sciences, Guangzhou, China; ^2^ The Second School of Clinical Medicine, Southern Medical University, Guangzhou, China; ^3^ Department of Stomatology, Zhuzhou Central Hospital, Xiangya School of Medicine, Central South University, Zhuzhou, China; ^4^ Botnar Research Centre, Nuffield Department of Orthopaedics, Rheumatology and Musculoskeletal Sciences, Medical Sciences Division, University of Oxford, Oxford, United Kingdom; ^5^ School of Medicine and Institute for Life Sciences, South China University of Technology, Guangzhou, China

**Keywords:** extracellular vesicle, bioengineering, biotechnology, tissue repairing, diagnosis, targeted therapy

Extracellular vesicles (EVs) are a type of nanoscale vesicles encapsulated by bilayer lipid membrane, which are mainly divided into several categories based on varied diameter and biological pathway: exosomes (Exos), microvesicles (MVs), and apoptotic bodies (ApoBDs) (EL Andaloussi et al., 2013). Almost all types of cells can secrete extracellular vesicles, which carry a variety of macromolecules such as nucleic acids (DNA, mRNA, microRNA, lncRNA), lipids, proteins, and metabolites, mediating intercellular communication. Native EVs can play a physiological role in promoting cell proliferation and regulating inflammation. However, due to their small production, low concentration, and high levels of impurities, native EVs have lower potential in therapeutic effects compared with engineered EVs. In this Research Topic, multiple studies have demonstrated the role and mechanism of engineered extracellular vesicles in promoting tissue healing and inflammatory regulation, and introduced the cutting-edge methods for preparing engineered extracellular vesicles.

In terms of the mechanism of tissue repairing by EVs, Lyu et al. demonstrated that M2-EXO enhanced the angiogenic ability of human umbilical vein endothelial cells (HUVECs) *in vitro* by transferring miR-21-5p, which inhibited PTEN expression and activated the AKT/mTOR pathway. Extensive research has shown that macrophages are closely related to angiogenesis at the wound site (Hesketh et al., 2017). Previous studies have mostly focused on the effect of M2 macrophages on wound healing, with few articles analyzing the role of M2-EXO, an extracellular vesicle secreted by M2 macrophages. In this study, the researchers used a full thickness cutaneous wound mice model to demonstrate that M2-EXO treatment promotes angiogenesis and accelerates skin healing. These findings highlight the promising therapeutic potential of M2-EXO as a cell-free method for enhancing skin healing and angiogenesis, which shows implications to translating into clinical use.

The study conducted by Wang et al. provides valuable insights into the potential of using extracellular vesicles derived from 4D culture (4D-sEVs) to reduce inflammatory response after spinal cord injury (SCI). Researchers obtained 4D-sEVs from human umbilical cord mesenchymal stem cells (hUC-MSCs) using 3D culture technology based on porous scaffolds. Proteomics analysis revealed significant changes in protein mass spectrometry, including the upregulation of Epidermal growth factor receptor (EGFR) and Insulin-like growth factor-binding protein 2 (IGFBP2) in 4D-sEV. *In vitro* and in SCI rats, 4D-sEV demonstrated the effective induction of macrophages/Microglia from pro-inflammatory M1 to anti-inflammatory M2 phenotype polarization. In addition, delivering 4D-sEV to the injured site leads to reduced neuroinflammation and increased tissue repair. This new method is expected to suppress the inflammatory response and promote recovery in SCI patients.

The structure and characteristics of EVs’ carrier can affect its effectiveness in tissue repairing. Xia et al. developed a photocrosslinked hydrogel capable of loading exosomes, which can promote hepatocyte regeneration after Hepatectomy. At present, the commonly used hemostasis methods of Hepatectomy include suture hemostasis, electrocoagulation, ultrasonic scalpel, etc. As a new postoperative hemostasis treatment, the light crosslinked adhesive hydrogel loaded with extracellular vesicles can promote hemostasis, accelerate cell proliferation and migration, and promote wound repair and tissue regeneration. The hydrogel is composed of modified gelatin matrix (GelMA) and alginate dopamine (Alg-DA). In order to further enhance its application in liver regeneration, adipose derived mesenchymal stem cell exosomes (AD-MSC-Exo) were loaded into GelMA/Alg-DA-1 hydrogel to improve cell proliferation and migration.

Hao et al. summarized the application and therapeutic mechanism of engineered stem cell extracellular vesicles in oral and maxillofacial wound healing. Through different methods such as genetic engineering, co incubation, parental cell surface modification, and artificial synthesis, stem cell extracellular vesicles with higher purity, targeting, and drug delivery efficiency can be engineered and prepared, achieving more significant therapeutic effects. Engineering stem cell exosomes can promote the healing of oral and maxillofacial wounds by regulating inflammatory reaction, promoting fibroblast proliferation, angiogenesis, and reducing the formation of scars, thus have a promising prospect for their application in Regenerative medicine.

Wei et al. reviewed the application of engineered extracellular vesicles in the treatment of degenerative orthopedic diseases. EV carries a variety of biologically active molecules, which can play an anti-inflammatory role, reduce chondrocyte apoptosis, and promote tissue repair (Silverman, 1986; Joseph et al., 2016; Lee et al., 2021; Mustonen and Nieminen, 2021). It has made certain research progress in the treatment of various DOD diseases such as OA, OP, and IDD. The engineering EVs typically have better performance and thermal potential than native EV. Currently, the main engineering methods include active loading and passive loading.

As a cell-free therapy, engineered extracellular vesicles have higher safety, lower immunogenicity and higher targeting, compared with stem cell therapy. In addition to the clinical applications mentioned above, EVs have also found application value in cardiac repair, fatty liver treatment and other fields (Wang et al., 2021; Pezzana et al., 2021). How to obtain therapeutic EVs with a well targeting property and good efficacy is the biggest challenge to promote its clinical application. EVs’ excellent ability in cell-growth promoting, anti-inflammatory and immune regulatory effects will widen its application in an increasing number of clinical fields.
